# Arthroscopic Anterior Cuciate Ligament Reconstruction Using Neither a Tourniquet nor Drainage: A Perioperative Case Series Report

**DOI:** 10.3390/life15040619

**Published:** 2025-04-07

**Authors:** Dimitrios A. Flevas, Michail Sarantis, Georgios Tsakotos, Grigorios G. Sasalos, Anastasios V. Tokis

**Affiliations:** 1 Arthroscopy and Orthopaedic Surgery Department, Metropolitan Hospital, Neo Faliro, 18547 Athens, Greece; 2 Anatomy Department, Medical School, National and Kapodistrian University of Athens, 10559 Athens, Greece

**Keywords:** arthroscopy, ACL reconstruction, no tourniquet, no drainage, arthroscopy without tourniquet

## Abstract

Introduction: Many orthopedic surgeons recommend ischemic tourniquets during arthroscopic anterior cruciate ligament (ACL) repair to reduce blood loss and improve visibility. However, their use remains controversial due to potential complications. Similarly, the practice of postoperative drainage is debated. While its proponents argue it reduces limb swelling, DVT, adhesions, and stiffness, others contend that it may increase infection risk or harm the ACL graft and joint surfaces. Materials and Methods: A total of 456 patients underwent anterior cruciate ligament reconstruction between September 2015 and December 2024, without the use of a tourniquet or drainage. The patients were 334 men with a mean age of 34.7 years and 122 women with a mean age of 32.3 years. In 389 cases the graft type was a hamstring autograft, in 55 cases a patellar tendon autograft (BPTB) was used, and in 12 cases a quadriceps tendon autograft was used. Results: The mean operative time was 61 min (range 52–79). No cases experienced visual impairment or required ischemia to enhance visibility. Bleeding sites were successfully cauterized during arthroscopy. Postoperative complications included knee hematoma in three patients (0.7%), resolved after drainage on day one, and two infections (0.4%), treated successfully with arthroscopic drainage and implant removal. No further complications were reported. Conclusion: Although many orthopedic surgeons prefer arthroscopic ACL repair with a tourniquet for better visibility and reduced intraoperative blood loss, this approach carries risks such as nerve palsy, joint swelling, stiffness, muscle weakness, and vascular changes. Not using a tourniquet can help to identify bleeding sites and allows for a more thorough procedure. The literature suggests that avoiding a tourniquet also reduces postoperative pain and accelerates recovery. The mean operative time for ACL reconstruction was consistent with the literature, indicating that avoiding a tourniquet did not cause delays. Additionally, the absence of postoperative drainage did not lead to complications, with most patients showing no issues like bleeding, hematoma, ischemia, or poor wound healing.

## 1. Introduction

With the rise in sports injuries, knee arthroscopic surgery has become a globally accepted minimally invasive and reliable surgical method [1,2,3]. The utilization of tourniquets in sports medicine, and especially arthroscopic anterior cruciate ligament (ACL) reconstruction, is widespread among orthopedic surgeons, aiming to reduce intraoperative blood loss, enhance surgical visualization, and achieve shorter operation times [1,4,5,6,7,8,9].

The tourniquet, a standard instrument in both arthroscopic and open knee surgeries, functions by managing bleeding and enhancing technical precision [1]. Consisting of a circular pneumatic cuff, it allows for high-pressure air inflation via a programmable pump. By inducing transient ischemia in the surgical area, the tourniquet aids surgeons in creating a bloodless operative field, facilitating a clearer visualization of anatomical structures. Additionally, the tourniquet serves as a hemostatic measure in cases of excessive bleeding [10,11].

Despite the advocacy for tourniquet usage in arthroscopic ACL reconstruction to enhance visibility and minimize blood loss, its application during knee arthroscopy remains debatable, with various studies and reviews examining its efficacy and associated risks. Notably, an increased risk of nerve palsy linked to tourniquet application has been highlighted by several authors [4,12,13]. Furthermore, as tourniquet utilization becomes more widespread, attention is directed towards other associated complications, including skin abrasions, blisters, compartment syndrome, neuromuscular damage, weakness of the quadriceps femoris, postoperative pain, subsequent blood loss, venous thrombosis, and pulmonary embolism [1,4,11,14,15,16,17].

Several studies in the past have investigated the omission of tourniquet usage during ACL reconstruction surgery, yielding conflicting outcomes [7,8,18,19]. Although pneumatic tourniquets were first introduced by Harvey Cushing in 1904 [20], advancements have led to reduced complications owing to precise pressure control. Nonetheless, the overall burden of tourniquet-related complications, encompassing systemic issues such as lactic acidosis, reperfusion injury, cardiovascular complications, muscular and vascular injuries, and nerve ischemia, is escalating with prolonged use [21,22,23].

The placement of surgical drains following orthopedic procedures has been a long-standing practice aimed at preventing hematoma formation, reducing pain and swelling, and facilitating early mobilization. Traditionally, drains have been expected to hasten the return of motion, shorten hospital stays, accelerate rehabilitation, and decrease the risk of infection. However, recent studies have questioned the necessity and efficacy of routine drain placement, particularly in joint arthroplasty, revision cases, and trauma surgeries. Some findings suggest that the presence of drains does not significantly impact postoperative clinical outcomes or the duration of hospital stays, leading to a reconsideration of their routine use [24].

The debate over the use of surgical drains extends to various arthroscopic procedures, particularly ACL reconstruction. Postoperative hemarthrosis remains a prevalent complication, occurring in up to 24% of cases, often leading to increased pain, stiffness, and delayed recovery. To mitigate these issues, surgeons commonly employ compression dressings and intra-articular drains. Advocates for drain placement argue that it prevents adhesions, reduces swelling, and minimizes joint stiffness, thereby enhancing functional recovery. Despite these potential advantages, critics raise concerns regarding the risks associated with drain placement. Increased susceptibility to infection, potential damage to intra-articular structures, and interference with graft healing are notable drawbacks. Additionally, some studies indicate that drains may not significantly reduce postoperative complications, questioning their overall benefit in arthroscopic procedures, particularly ACL reconstruction [25,26].

With this retrospective study, we attempt to present our experience in arthroscopic ACL reconstruction without the application of tourniquet or drains.

## 2. Materials and Methods

This study was conducted between September 2015 and December 2024, during which a total of 456 consecutive adult patients underwent anterior cruciate ligament (ACL) reconstruction without the application of a tourniquet or postoperative drainage. Among the 456 patients included in this study, 334 were male and 122 were female. The mean age of the male patients was 34.7 years (ranging from 18 to 53 years), while the mean age of the female patients was 32.3 years (ranging from 18 to 47 years) (Table 1). Patients were selected based on their indication for ACL reconstruction, which was determined by clinical evaluation, the MRI confirmation of ligament rupture, and functional impairment associated with instability of the knee joint. Exclusion criteria included previous knee infections, severe osteoarthritis, coagulation disorders, and a history of multiple ligament injuries requiring additional surgical intervention.

The choice of graft used for ACL reconstruction was based on surgeon preference, patient-specific anatomical considerations, and preoperative discussions regarding expected functional outcomes. Among all the cases, 389 patients received a hamstring tendon autograft. More specifically, a quadrupled two-tendon hamstring autograft was used in 339 cases, while a semitendinosus tendon graft was utilized with the all-inside technique in 50 cases (34 males, 16 females). A total of 55 patients underwent ACL reconstruction using a bone–patellar tendon–bone (BPTB) autograft, selected primarily for its superior fixation properties and lower risk of elongation postoperatively. In total, 12 patients received a quadriceps tendon autograft, an emerging alternative providing excellent biomechanical strength (Table 1).

The surgical procedure was performed under spinal or general anesthesia, based on patient preference and anesthesiologist recommendation. A standard arthroscopic technique was used for ACL reconstruction. The femoral and tibial tunnels were prepared according to the respective graft choices, ensuring appropriate positioning to restore knee kinematics.

**Figure 1:**
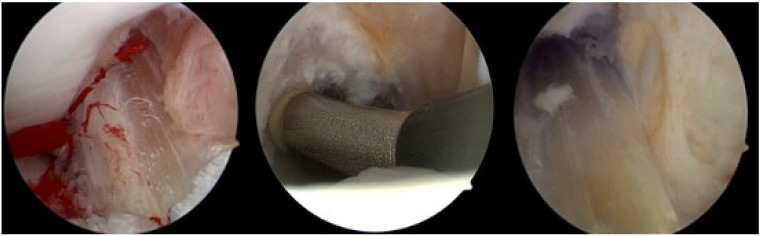
Direct identification of a bleeding point and immediate coagulation with subsequent improvement of visualization.

A key component of this study was the elimination of a tourniquet during surgery. Instead, in all cases, an automated volumetric pump was used to maintain joint distension and control bleeding, ensuring adequate visualization throughout the procedure. Additionally, without the use of a tourniquet, bleeding points could be identified, allowing for immediate coagulation using a bipolar arthroscopic RF system (Figure 1). Furthermore, an intra-articular injection of tranexamic acid (TXA) was administered to further reduce intraoperative and postoperative bleeding.

All the patients followed a standardized postoperative protocol, including early mobilization with partial weight-bearing using crutches for the first two weeks. Physiotherapy was initiated immediately, focusing on reducing swelling, restoring range of motion, and strengthening the quadriceps and hamstring muscles. Patients were advised to use cold therapy and compression to manage swelling and discomfort.

## 3. Results

The mean operative time for ACL reconstruction in this study was 61 min, with a range of 52 to 79 min, and all patients were discharged the day after the operation as standard practice. Notably, the absence of a tourniquet did not compromise intraoperative visibility in any case. Bleeding was effectively managed using arthroscopic cautery and the intra-articular injection of TXA, eliminating the need for a tourniquet. The automated volumetric pump provided sufficient joint distension, allowing surgeons to perform procedures without any impairment or diminished visualization.

Among the 456 patients, no one reported thigh pain or neurological deficits after surgery, as these complications are typically associated with tourniquet use. Overall, the incidence of postoperative complications was low.

Three patients (3/456, 0.7%) developed knee hematomas requiring aspiration on the first postoperative day (Table 2). These patients exhibited localized swelling and discomfort, which were effectively resolved through a single aspiration procedure. No further interventions were necessary, and they proceeded with the standard rehabilitation protocol without additional complications. No patient required an additional day of hospitalization, not even those who developed hematomas, as they were treated immediately and discharged accordingly.

Two patients (2/456, 0.4%) developed postoperative infections (Table 2), presenting with pain and swelling within 20 days of surgery. Both cases required arthroscopic drainage and implant removal, followed by a course of intravenous antibiotics. Subsequent follow-up revealed the successful resolution of the infection.

No other major complications, such as deep vein thrombosis, neurovascular injury, graft failure, or stiffness requiring manipulation under anesthesia, were observed in the study cohort.

## 4. Discussion

This retrospective study examines the outcomes of a substantial number of arthroscopic ACL reconstructions performed without the use of a tourniquet or drain. The perioperative results strongly support this practice, as the incidence of complications related to the omission of a tourniquet or drain is minimal, and any complications observed cannot definitively be attributed to this factor. These findings align with a growing body of literature that questions the necessity of tourniquet use in knee arthroscopy and highlights the potential benefits of avoiding both tourniquets and postoperative drainage systems.

The use of tourniquets in arthroscopic ACL reconstruction is a widespread practice among orthopedic surgeons, primarily aimed at reducing intraoperative blood loss, improving surgical visualization, and potentially reducing operation times [4,5,6,7,8,11,14]. Theoretically, improved visualization allows for more precise graft placement, potentially enhancing surgical outcomes. However, some studies have suggested that tourniquet use does not significantly impact visualization or operative time in knee arthroscopic surgery [1,27], while others have found that avoiding tourniquet use may facilitate hemostasis and reduce postoperative pain [1,28].

Tourniquet application has been associated with various complications, including increased postoperative pain [28], early infection, wound healing disorders, swelling, joint stiffness, and vascular changes leading to deep vein thrombosis (DVT) and pulmonary embolism (PE) [1,4,11,14,15,16,17,23]. These risks raise concerns about the routine use of tourniquets, particularly when alternative strategies for controlling intraoperative bleeding, such as tranexamic acid (TXA) administration and an automated volumetric pump, are available. Additionally, an elevated risk of nerve palsy has even been noticed in some cases [12,13].

A systematic review and meta-analysis in 2024 suggested that using a tourniquet during arthroscopic ACL reconstruction leads to increased postoperative drain output, higher pain intensity after 22 h, and a reduction in thigh girth compared to procedures performed without a tourniquet. These results indicate that avoiding a tourniquet may enhance early postoperative outcomes by minimizing patient discomfort and postoperative swelling. Additionally, the absence of a significant effect on operation time when forgoing the tourniquet suggests that surgical efficiency remains unaffected. These findings support the consideration of non-tourniquet techniques in ACL reconstruction to improve patient recovery [28]. Researchers have suggested that the application of a tourniquet might delay axonal conduction, leading to heightened postoperative pain and ultimately resulting in nerve paralysis [4,29]. Given these potential risks, our study reinforces the idea that omitting a tourniquet may be a viable and potentially advantageous approach to ACL reconstruction.

The efficacy of tourniquet use in ACL reconstruction remains a subject of debate, with conflicting findings regarding its impact on patient outcomes. Hoogeslag et al. suggested that tourniquet use resulted in better arthroscopic visualization but did not improve operative time [30], and Zaid et al. advocated the use of a tourniquet during anterior cruciate ligament reconstruction for enhancing intraoperative visualization, reducing operative time, and minimizing intraoperative sterile saline consumption and blood loss, without leading to serious adverse events or increased complication rates in early postoperative outcomes [31]. Conversely, Daniel et al. reported a delayed recovery of quadriceps femoris strength with tourniquet application at 12 weeks postoperation but found no significant difference at 52 weeks [18]. Similarly, Nicholas et al. observed a decrease in thigh circumference at three weeks with tourniquet use but no difference in muscle strength at six months [32]. Nakayama et al. found no functional difference between groups three months postoperation [19] while Su et al. reported that tourniquet use for arthroscopic meniscal repair does not affect primary outcome or secondary outcomes [33]. Regarding the impact of tourniquet application on the skin, a prospective randomized trial conducted by Din and Geddes suggested that lower limb tourniquets should be accompanied by additional skin protection underneath to prevent tissue damage [15].

Given the mixed results in the existing literature, our study contributes to the growing evidence that ACL reconstruction can be performed effectively without a tourniquet, with comparable or even improved patient outcomes. The low complication rate observed further supports this approach.

The use of postoperative intra-articular drains following arthroscopic ACL reconstruction is another topic of ongoing debate. Theoretical advantages of drainage include the removal of serous fluids deficient in opsonic proteins, which are crucial for immune function, and the prevention of hematoma accumulation, a potential medium for bacterial growth. Closed-suction drainage systems aim to mitigate contamination risk, yet they may inadvertently increase bleeding in drained wounds and pose risks if dysfunctional. Additionally, there is always the potential for inadvertent damage to the articular cartilage or the graft during drain placement [25,26,34]. The necessity of drains has been evaluated in various studies, with mixed findings. McCormack et al. found no significant difference in pain and range of motion outcomes between drain and non-drain groups in 118 arthroscopic ACL reconstruction patients [35]. Khalifa et al. randomized forty patients into drain and non-drain groups, observing lower hemarthrosis grades in the drain group and fewer aspirations required within the first two days post-surgery compared to the non-drain group. However, no hemarthrosis cases developed in either group over the follow-up period [36].

The effect of drains on early postoperative pain remains controversial in prior randomized trials. A study by Shimozaki et al. found that intra-articular drain placement after ACL reconstruction did not impact postoperative pain, knee range of motion, muscle strength at three months, or complication rates. However, pain levels at four hours post-surgery were lower in the non-drain group compared to the drain group, suggesting limited benefits of intra-articular drain placement. In this study, the drain group experienced higher pain levels at four hours post-surgery, aligning with previous research [24]. This can be attributed to improper drain placement or incorrect length, which can cause displacement into the suprapatellar pouch. Such misplacement may lead to trapping symptoms and pain due to suction irritation of the synovial tissue, particularly with minor knee movements that pinch the patellofemoral joint. However, it seems arbitrary to attribute all pain symptoms to improper drain placement.

In our study, no immediate complications related to the absence of a drain were observed. The incidence of hematomas requiring treatment was exceedingly low (0.07%), and it cannot be definitively attributed solely to the absence of a drain. This supports the growing notion that the routine use of postoperative drains may be unnecessary and that their risks may outweigh their benefits.

There are several limitations to consider regarding the present study. The major limitation is the lack of a control group, which prevents direct comparisons between tourniquet and non-tourniquet groups, as well as between drain and non-drain groups. However, this study was conducted as a retrospective observational study with a perioperative descriptive character, providing valuable insights into the feasibility of tourniquet- and drain-free ACL reconstruction. Another limitation is the lack of an objective functional outcome score to quantitatively assess patient recovery and knee function over time. Although standard clinical evaluations and patient-reported outcomes were utilized, a more systematic approach incorporating objective scoring systems would strengthen the findings. Additionally, the uneven follow-up time across patients introduces variability, which may affect the long-term interpretation of our results.

## 5. Conclusions

The use of a tourniquet during arthroscopic anterior cruciate ligament (ACL) reconstruction is a common practice among orthopedic surgeons, primarily aimed at reducing intraoperative blood loss and improving visibility. However, this approach is not without complications, since tourniquet application has been associated with an increased risk of nerve palsy, postoperative swelling, joint stiffness, decreased muscle endurance, functional weakness, electromyographic alterations, and vascular changes.

Our study demonstrates that arthroscopic ACL reconstruction can be safely and effectively performed without the use of a tourniquet or a postoperative drainage system. Intraoperative bleeding was successfully managed using alternative techniques such as an automated volumetric pump and intra-articular tranexamic acid (TXA) administration, without compromising surgical visualization or efficiency. Furthermore, avoiding tourniquet-related complications—such as increased postoperative pain, nerve injuries, and delayed quadriceps function recovery—may enhance overall patient outcomes and expedite rehabilitation.

Similarly, the absence of postoperative drainage did not lead to a significant increase in complications, reinforcing the notion that routine drainage may not be necessary following ACL reconstruction. Our findings suggest that eliminating both tourniquet use and postoperative drainage may contribute to improved patient comfort and recovery without negatively affecting surgical outcomes. However, our study is limited by its retrospective design and lack of a control group. Although the low complication rates that were observed support the safety and efficacy of this approach, future randomized controlled trials with larger sample sizes, longer follow-up periods, and standardized outcome measures are necessary to validate these findings. Further research will help to refine surgical techniques and optimize patient care, ensuring the best possible outcomes in ACL reconstruction.

## Figures and Tables

**Table 1:** Patients’ demographics and surgical details.

	N	Mean Age (Range)	Hamstrings	BPTB	QT	Knee Hematoma	Infection
Male	334	34.7 (18–53)	282	40	12	2	2
Female	122	32.3 (18–47)	107	10	5	1	
Total	456		389	50	17	3	2

BPTB: Bone-Patellar Tendon-Bone, QT: Quadriceps Tendon.

**Table 2:** Demographics and surgical details of complicated patients.

Type of Complication	N	Mean Age (Ages)	Male	Female	Hamstrings
Knee Hematoma	3	28 (24, 29, 31)	2	1	3
Infection	2	48 (46, 50)	2	-	2

## Data Availability

The data presented in this study are available on request from the corresponding author. The data are not publicly available.
